# Interleukin-22 Induces the Infiltration of Visceral Fat Tissue by a Discrete Subset of Duffy Antigen Receptor for Chemokine-Positive M2-Like Macrophages in Response to a High Fat Diet

**DOI:** 10.3390/cells8121587

**Published:** 2019-12-06

**Authors:** Eun-Young Kim, Hye Mi Noh, Bongkun Choi, Ji-Eun Park, Ji-Eun Kim, Youngsaeng Jang, Hyung Keun Lee, Eun-Ju Chang

**Affiliations:** 1 Department of Biomedical Sciences, Asan Medical Center, University of Ulsan College of Medicine, Seoul 05505, Korea; kimberly_kim44@hotmail.com (E.-Y.K.); bkchoi89@hanmail.net (B.C.); hjku2020@naver.com (J.-E.P.); kge33@sookmyung.ac.kr (J.-E.K.); choib01@naver.com (Y.J.); 2 Stem Cell Immunomodulation Research Center, Asan Medical Center, University of Ulsan College of Medicine, Seoul 05505, Korea; 3 Department of Ophthalmology, Institute of Vision Research, Yonsei University College of Medicine, Seoul 06273, Korea; hmnoh@kpclab.co.kr; 4 Department of Pharmacy, Integrated Science and Engineering Division, Yonsei University, Incheon 21983, Korea

**Keywords:** interleukin 22, monocyte-derived macrophages, Duffy antigen receptor for chemokines (DARC), adipose tissue, inflammation

## Abstract

Interleukin-22 (IL-22) is a cytokine with important functions in host defense and inflammatory responses and has recently been suggested to play a role in immune-inflammatory system in the context of obesity and its metabolic consequences. The specific cellular targets and mechanisms of IL-22-mediated obesity are largely unknown however. We here identified a previously unknown subset of monocyte-derived Duffy antigen receptors for chemokines (DARC)^+^ macrophages in epididymal fat adipose tissue and found that they are preferentially recruited into the crown-like structures of adipose tissue in the mouse upon high fat diet-induced obesity. Importantly, DARC^+^ macrophages highly express the IL-22 receptor (IL-22Ra1). Exposure to recombinant IL-22 shifts macrophages to an alternative M2 polarization pathway and augments DARC expression via a STAT5b signaling axis. STAT5b directly binds to the DARC promoter and a STAT5 inhibitor abrogates the IL-22-mediated induction of DARC. These M2-like DARC^+^ subpopulations of monocytes/macrophages were elevated in obese db/db mice compared to WT lean mice. Furthermore, subsets of CD14^+^ and/or CD16^+^ monocytes/macrophages within human peripheral blood mononuclear cell populations express DARC and the prevalence of these subsets is enhanced by IL-22 stimuli. This suggested that IL-22 is a critical cytokine that promotes the infiltration of adipose tissue macrophages, that regulate inflammatory processes. Taken together, our present findings provide important insights into the molecular mechanism by which IL-22 signal modulates DARC expression in M2-like macrophages.

## 1. Introduction

Adipose tissue is involved in various biological functions including storage of excess energy, cold insulation, and the mechanical protection of the vital organs. In addition, the adipose tissue produces and secretes a variety of cytokines, known as adipokines, principally from the immune cells in fat and thus functions as an endocrine organ for regulating immune-inflammatory responses [1,2]. Adipose tissue is therefore not only an energy source via fat storage but also an active part of the immune system that regulates the inflammatory responses of the body [3]. The adipose tissue contains various types of cells including immune cells (e.g., macrophages, dendritic cells, B and T cells) in addition to adipocytes [4,5,6,7,8]. Macrophages are one of the most abundant cell types in adipose tissue and thus have been extensively studied as important contributors to the function of adipose tissue. Macrophages are unique in their capacity to quickly adapt to a changing environment. These cells make up around 10% of the total cells in white fat tissue in lean mice and humans, whereas they constitute up to 50% of the total in extremely obese and leptin-deficient mice and almost 40% in obese human subjects [1,9]. Adipose tissue macrophages (ATMs) increase their cell number and change their phenotype such as localization and inflammatory features during obesity. ATMs are localized throughout the adipose tissue and display limited pro-inflammatory features in the lean condition. In contrast, ATMs in obese state accumulate around dead adipocytes to form crown-like structures (CLS) and produce abundant proinflammatory cytokines [10,11,12]. Moreover, diet-induced obesity leads to a shift in the activation state of ATMs from an alternative M2-polarized state to a classical M1 proinflammatory state [10,13]. Despite the extensive investigations of ATMs however, their mechanism of generation, infiltration into other tissues, cross-talk with adipocytes, and functional role in obesity are still controversial [14,15,16].

Obesity is a state of chronic low-grade systemic inflammation which induces the production of several chemokines including CCL2, CCL5, and CXCL5 [17,18]. The CCL2-CCR2 axis is of central mediator that promotes ATM recruitment [19,20]. The atypical chemokine receptor 1 (ACKR1), also known as the Duffy antigen receptor for chemokines (DARC), exhibits binding capacity for a wide variety of C-C and C-X-C chemokines and is expressed in erythrocytes, cerebellar Purkinje cells, and endothelial cells [21,22]. DARC is a seven-transmembrane protein which does not activate G protein coupled signaling pathways because of a lack of the triplet sequence Asp-Arg-Tyr (DRY motif) in its second intracellular loop [23]. However, the erythrocyte DARC may also serve as a blood reservoir by sequestering chemokines [24] and the endothelial cell DARC can mediate chemokine transcytosis [25]. Recently, Benson et al. reported that a DARC deficiency causes augmented adipose tissue inflammation, as evidenced by increased CCL2 level accompanied by abundant macrophage CLSs in adipose tissue leading to induction of glucose intolerance [26]. This indicated the possible involvement of DARC in metabolic disorders.

Interleukin 22 (IL-22) is a recently identified small protein which has various functions, including the regulation of inflammatory responses, coordination between innate and adaptive immunity, modulation of metabolic alterations, epithelial cell integrity, and cell survival [27,28,29]. Given the connection between altered gut microbiome composition, chronic inflammation, and metabolic disorders, IL-22 has been postulated to contribute to the regulation of metabolic functions via the modulation of mucosal immunity [28,29]. Additionally, under a homeostatic status, IL-22 is unambiguously produced by immune cells such as T cells, NK cells, and ILC3 and binds to IL-22 receptor-1^+^ (IL-22R1^+^) [30]. However, in a pathologic state, the exact cellular sources of IL-22 in human patients with disease are not yet defined and are presumed to vary depending on the nature of the disorder [27]. Moreover, little is known about the role of IL-22 in ATM proliferation in the bone marrow (BM) and the infiltration of these cells into the fat tissue. Beside macrophages, there has been a growing emphasis on T cell populations, which vary in proportion and phenotype in obesity. CD4^+^ Th cells and CD8^+^ cells with pro- or anti-inflammatory cells were found to accumulate in the adipose tissue of obese mice [7,31,32]. The accumulation of IL-17- and/or IL-22-producing T cells is also evident in the human adipose tissue of insulin-resistant obese patients [31], raising the possibility that IL-22 may be involved in obesity-mediated inflammation. However, the specific cellular targets and mechanisms of IL-22-mediated obesity remain largely unknown. Hence, a deeper understanding of the IL-22-mediated regulation of ATM, and more refined phenotype and functional characterizations will help to delineate IL-22-mediated obesity.

In our current study, we provide the first evidence of a rare population of monocyte-derived M2-like DARC^+^ macrophages that reside in the epididymal fat adipose tissues in mouse and peripheral blood mononuclear cells (PBMCs) in human subjects. We further found an augmented recruitment of these cells into the adipose tissue of crown-like structures in a diet-induced obesity murine model. Notably, DARC^+^ macrophages highly expressed IL-22Ra1, which is rarely expressed in the myeloid cell lineage, and IL-22 was further found to induce DARC expression. In addition, we show from our current analyses that the induction of DARC through the “IL-22-IL-22Ra1-STAT5-linked axis” is important for maintaining the M2-like macrophage potentials in both mouse and human monocytes. Taken together, our present findings provide important insights into the molecular mechanism by which IL-22 signals regulate DARC expression in M2-like macrophages.

## 2. Materials and Methods

### 2.1. Human Samples

The cross sectional, case control human study was performed at Yonsei University College of Medicine, Seoul, Korea. All human subjects were treated according to the Declaration of Helsinki. Under protocol approved by the Institutional Review Board (3-2017-0035), all human subjects provided informed consent. PBMCs from healthy subjects (body mass index < 23 kg/m^2^; *n* = 8) were utilized for flow cytometry analysis.

### 2.2. Animal Experiments

All mouse studies were conducted according to the protocol approved by the Institutional Committee for the Care and Use of Laboratory animals of Ulsan University (2016-13315) and Yonsei University College of Medicine (2013-14478). C57BL/6J and C57BL/KsJ-db/db mice were purchased from Jackson Laboratory (Bar Harbor, ME, USA) and IL-22 KO mice (B6;129S5-Il22tm1Lex/Mmucd) were obtained from UC Davis MMRRC (Davis, CA, USA). After a minimum 1-week stabilization period, 7 weeks old male or female mice were fed with either standard pelleted chow (13% kcal from fat) or HFD (60% kcal from fat). After 12 weeks of HFD feeding, the animals were sacrificed. Portions of white adipose tissues from epididymal fat pads or spleen were fixed in 4% paraformaldehyde and embedded in paraffin or were further processed for splenic cells and SVC isolation for FACS analysis.

### 2.3. Experimental Reagents and Cell Cultures

Human recombinant IL-22 was obtained from R&D systems (Minneapolis, MN, USA). STAT5 inhibitor (STAT5i), CAS285989 was purchased from STEMCELL Technologies (Vancouver, BC, Canada). Fetal bovine serum (FBS) and non-essential amino acids were sourced from Life Technologies (Gaithersburg, MD, USA). All other chemicals were obtained from standard sources and were of molecular biology grade or higher. The human monocytic cell line, THP-1, and HEK293 cells were purchased from the American Type Culture Collection (Rockville, MD, USA) and maintained in RPMI 1640 medium (GIBCO^®^, Grand Island, NY, USA) with 10% FBS and antibiotic–antimycotic solution (Life Technologies) at 37 °C in a humidified atmosphere containing 5% CO_2_

### 2.4. Flow Cytometry (FACS)

The mouse spleens were digested with 1 mg/mL collagenase I (Gibco) in Hank’s balanced salt solution (HBSS; Life Technologies) and stained. The bone marrow (BM) was prepared from femur and BD Pharm Lyse (BD Biosciences) was added to lyse red blood cells. TruStain FcX antibody (BioLegend, San Diego, CA) was applied to block non-specific binding for 10 min at 4 °C in FACS buffer (Ca^2+^/Mg^2+^-free PBS with 1% human bovine serum albumin, 4% FBS, and 0.5 M EDTA) before staining (30 min) with appropriate antibodies. For intracellular staining, SVCs isolated from epididymal white adipose tissue (eWAT) were stimulated for 5 h at 37 °C with PMA, ionomycin, and GolgiStop (BD). Stimulated cells were washed with PBS, fixed, and permeabilized by Cytofix/Cytoperm kit (BD) as per the manufacturer’s protocol. Abs were purchased from BioLegend or R&D Systems: For mouse, CD45R/B220 (30-F11), F4/80 (BM8), Ly-6C (HK1.4), Ly-6G (1A8), CD3 (17A2), CCR7 (4B12), CD8a (53-6.7), CD11b (FAB1124S), CD4 (FAB554S), DARC (FAB6695A), CD206 (C068C2), IL-22Ra1 (FAB42941P), IL-22 (Poly5164), and IL-10 (JES5-16E3); for human, CD4 (RPA-T4), CD8 (SK1), CD14 (63D3), CD11b (ICRF44), CD16 (3G8), DARC (Clone #358307), IL-22Ra1 (Clone #305405), CD86 (IT2.2), and CD206 (15-2). Isotype control forward- and side-scatter parameters were used to remove the cell aggregates and debris.

### 2.5. Cell Sorting

For analysis of the DARC^+^ subset, human THP-1 cells, primary PBMCs isolated from human blood, or bone marrow cells from 8-week-old female C57BL/6J mice were stimulated for 24 h with 20 or 40 ng/mL of IL-22. CD14^+^ monocytes (for human), monocytes (CD11b^+^), and macrophages (F4/80^+^) (for mouse) were then sorted for expression analysis. Zombie NIR™ Fixable Viability kit (Biolegend) was used to exclude cell debris and dead cells. Sorting was carried out on a BD FACSCanto II (BD Biosciences) and >90% of the target population was obtained.

### 2.6. Isolation of SVCs From Epididymal White Adipose Tissue

Epididymal white adipose tissue (eWAT) was harvested from mice and SVCs were isolated by enzymatic digestion (collagenase II; Gibco). The digested tissue was filtered through a 100-μm mesh filter to remove debris. The cellular pellet containing the SVCs was resuspended with an ammonium chloride lysis buffer to remove red blood cells and then subjected to FACS analysis

### 2.7. RNA Extraction, RT-PCR, and Quantitative Real-Time PCR (qPCR)

Total RNA was isolated from mouse white adipose tissues or human THP-1 cells with Qiazol reagent (Invitrogen Life Technologies) following the manufacturer’s protocol. First-strand cDNA was synthesized from total RNA with RevertAid First strand cDNA Synthesis kit (Thermo Scientific) and cDNA was amplified in a BIO-RAD T100™-Thermal Cycler (Bio-Rad, Hercules, CA, USA). qPCR was carried out on the Light Cycler 480 Real time-PCR Detection system (Roche) using SYBR Green PCR master mix (Roche, Penzberg, Germany) according to the manufacturer’s protocol. The PCR primers used are described in Table S1. Results are represented as the ratio of target PCR products relative to GAPDH internal control product. The relative quantities of mRNA were calculated utilizing the ΔΔCT method.

### 2.8. Luciferase Assay

HEK 293 cells were seeded onto 12-well plates at 1 day before transfection. At 40–50% confluency, 500 ng of luciferase reporter construct (wild-type, mutant M1, M2, or M3 stat5b binding site including core promoter of *ackr1*) and 100 ng of Renilla luciferase construct were transfected with Lipofectamine 2000 (Life Technologies) into the cells for 6 h. After 24 h, cells were stimulated with 20 ng/mL of IL-22 for 24 h, and then lysed. Luciferase activity was determined utilizing the Dual Luciferase Reporter Assay System (Promega) in accordance with the manufacturer’s protocols.

### 2.9. Western Blotting Analysis

THP-1 cells or cells transfected using the Gene Pulser Xcell™ Electroporation Systems (Bio-Rad) were lysed in RIPA buffer (50 mM Tris/HCl [pH 7.4], 0.25% sodium deoxycholate, 1% Nonidet P40, and 150 mM NaCl) in the presence of phosphatase and protease inhibitors. The protein lysates were resolved using SDS–PAGE and electophoretically transferred to polyvinylidene difluoride (PVDF) membranes (Bio-Rad, Hercules, CA, USA). Each membrane was blocked using 5% bovine serum albumin solution for 1 h and probed with anti-DARC (NBP1-77278, Novus Biologicals) or anti-β-actin (A5441, Sigma) overnight at 4 °C. Bound antibodies were visualized with the corresponding secondary antibodies conjugated with HRP and an enhanced chemiluminescence Western blotting system (Merck-Millipore). 

### 2.10. Chromatin Immunoprecipitation

ChIP assay with α-STAT5b Ab (Cell Signaling Technology, Danver, MA, USA) was performed as previously reported [33]. The predicted three *Ackr1* promoter sequence (−10,000~−4500) was amplified using the following primers: for PCR1 (−8830) (forward) 5′-ggctgcaaactgttgtcgt-3′, (reverse) 5′-cacccagagaggctgttcttt-3′, for PCR2 (−7180) (forward) 5′- gcttgtactatgccaagagacag-3′, (reverse) 5′-tttccgcagctcaagaagtg-3′, and for PCR3 (−5080) (forward) 5′-cctgttggactgcgtctgtt-3′, (reverse) 5′-tctcagggagtatgcgagaca-3′.

### 2.11. Immunohistochemical Analysis

Serial 5-µm-thick sections were cut through tissues from chow diet- or HFD-fed mice and immunohistochemical staining analysis with DARC antibodies (Abcam) was performed utilizing the REALTM EnVisionTM Detection System Peroxidase/DAB+ kit (Dako) according to the manufacturer’s instructions. Tissues were examined and photographed with an Olympus BX51 microscope (Olympus). 

### 2.12. Statistical Analyis

**Figure 1:**
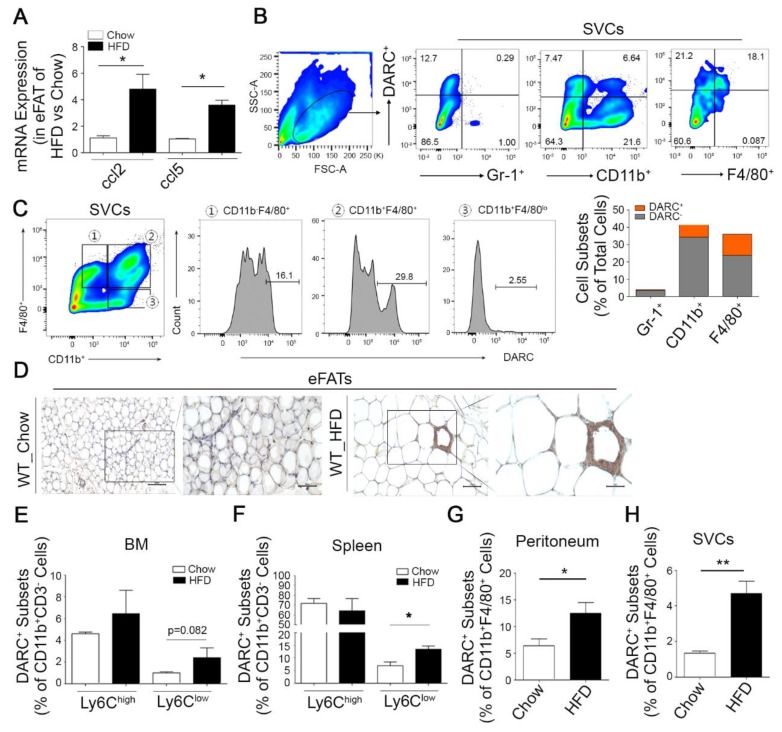
Identification of a distinct monocyte-derived Duffy antigen receptor for chemokines (DARC)^+^ macrophage subset in epididymal fat adipose tissues and increased infiltration of these DARC^+^ macrophages in mice fed with a high fat diet (HFD). (**A**) Increased expression of ccl2 and ccl5 was observed by qPCR in epididymal white adipose tissue (eWAT) from mice fed with an HFD (60 kcal from fat) for 12 weeks compared to mice fed with a normal diet (chow) (*n* = 4 mice per group). (**B**) Frequency of DARC^+^ subsets in the monocyte or macrophage populations in stromal vascular cells (SVCs) from eWATs of wild-type (WT) mice. Fluorescence-activated cell sorting (FACS) plots identified DARC^+^ subsets in populations of myeloid cells with Gr-1^+^ (left), CD11b^+^ (middle), or F4/80^+^ (right) markers relative to isotype control and the graph (lower) shows the relative proportions of DARC^+^ or DARC^-^ in eWAT monocytes/macrophages defined by Gr-1^+^, CD11b^+^, or F4/80^+^. Numbers inside indicate the percentage of each subset among the total SVCs. FSC, forward scatter; SSC, side scatter. (**C**) Myeloid subsets of SVCs were further gated as (1) CD11b^lo^F4/80^hi^, (2) CD11b^hi^F4/80^hi^, or (3) CD11^hi^F4/80^lo^ populations (left) and the frequencies of DARC^+^ monocytes/macrophages were analyzed in the corresponding histograms (right) relative to isotype control (Figure S2). Numbers above the bracketed lines (right) indicate the percentage of DARC^+^ cells. (**D**) C57BL/6J mice were fed with chow or HFD for 12 weeks, epididymal adipose tissue were isolated, and DARC protein expression was determined by immunohistochemical analysis (IHC). Representative IHC photomicrographs are shown. Scale bar, 100 μm (**D**, left), 50 μm (**D**, right). (**E**–**H**) Monocyte-derived DARC^+^ macrophages were generated from bone marrow (BM) cells and the number of these cells was increased in the HFD-induced obese mice. The abundance of DARC^+^ subsets, shown as a percentage of the total monocytes, was defined by CD11b^+^ and LyC detection in BM (**E**), spleen (**F**), peritoneum (**G**), or SVC fraction of eWATs (**H**) from mice fed with either a chow diet or an HFD (*n* = 5~6 mice per group). * *p* < 0.05, ** *p* < 0.01 versus chow. Values are expressed as a mean ± SD. Comparisons were performed with *t*-tests (two groups) or ANOVA (multiple groups). Data were combined from at least three independent experiments.

**Figure 2:**
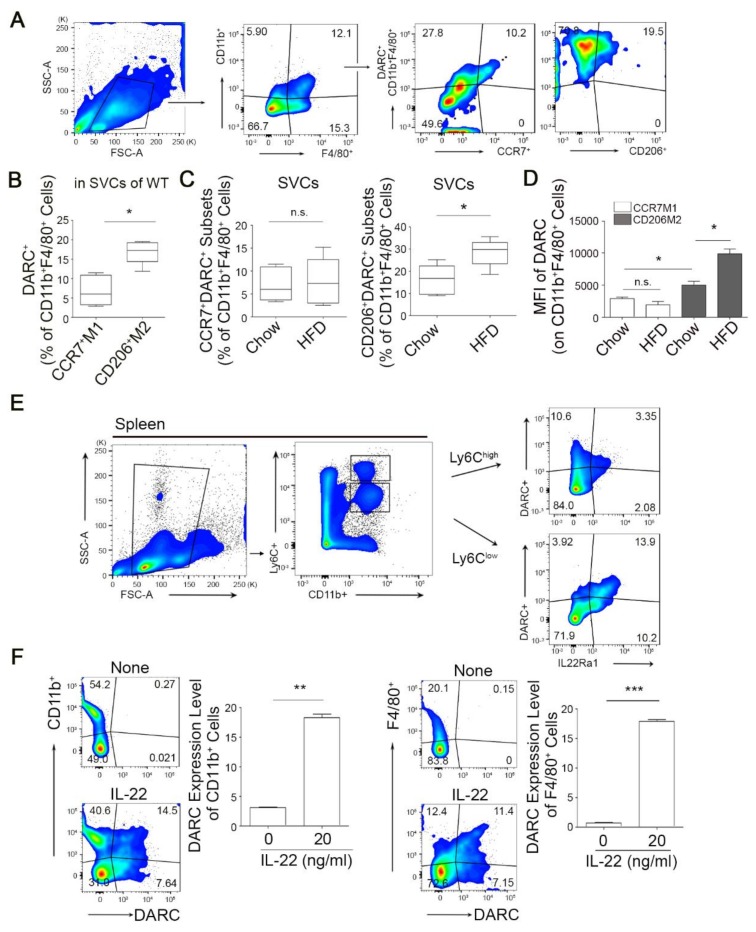
Increased infiltration of M2-like DARC^+^ macrophages following HFD and interleukin 22 (IL-22)-induced DARC expression. (**A**,**B**) Infiltrating monocyte-derived DARC^+^ cells show characteristics of M2-like macrophages. Frequency of DARC^+^ cells in CCR7^+^CD11b^+^F4/80^+^ (classical inflammatory M1) or CD206^+^CD11b^+^F4/80^+^ (alternative tissue repair M2) monocytes from CD11b^+^F4/80^+^ subset among the SVCs of wild-type adipose tissue (**A**) and these population were quantified (**B**). (**C**) Augmentation of the DARC^+^CD206^+^ subset, but not the DARC^+^CCR7^+^ subset, was identified in obese SVCs as shown by FACS analysis (*n* = 7~8 mice per group). * *p* < 0.05 versus the corresponding control. Data are shown as a mean ± SEM. Comparisons were performed using *t*-tests (two groups). Data were combined from at least three independent experiments. (**D**) Mean fluorescence intensity (MFI) of M2 DARC^+^ macrophages were increased after HFD consumption. MFI of DARC was determined in CCR7^+^CD11b^+^F4/80^+^ or CD206^+^CD11b^+^F4/80^+^ subset of chow- and HFD-fed mice. (**E**) Identification of IL22Ra^+^ cells in the DARC^+^ subset of splenic monocytes. The gating and staining strategies used in the FACS quantification of an IL-22Ra1^+^ subset in DARC^+^CD11b^+^Ly6C^lo^ or DARC^+^CD11b^+^Ly6^hi^ cells are shown. Numbers (black outline at left) indicate the percentage of CD11b^+^ cells among the splenocytes. (**F**) DARC expression was increased by IL-22 treatment of monocyte/macrophages. BM cells isolated from WT mice were treated with or without mIL-22 (20 ng/mL) for 24 h and the frequency of DARC^+^ cells among the CD11b^+^ or F4/80^+^ monocytes was then analyzed by FACS as described in the materials and methods. ** *p* < 0.01, *** *p* < 0.001 versus the control. Data were combined from at least three independent experiments and are shown as the mean ± SEM. Comparisons were performed using *t*-tests (two groups).

**Figure 3:**
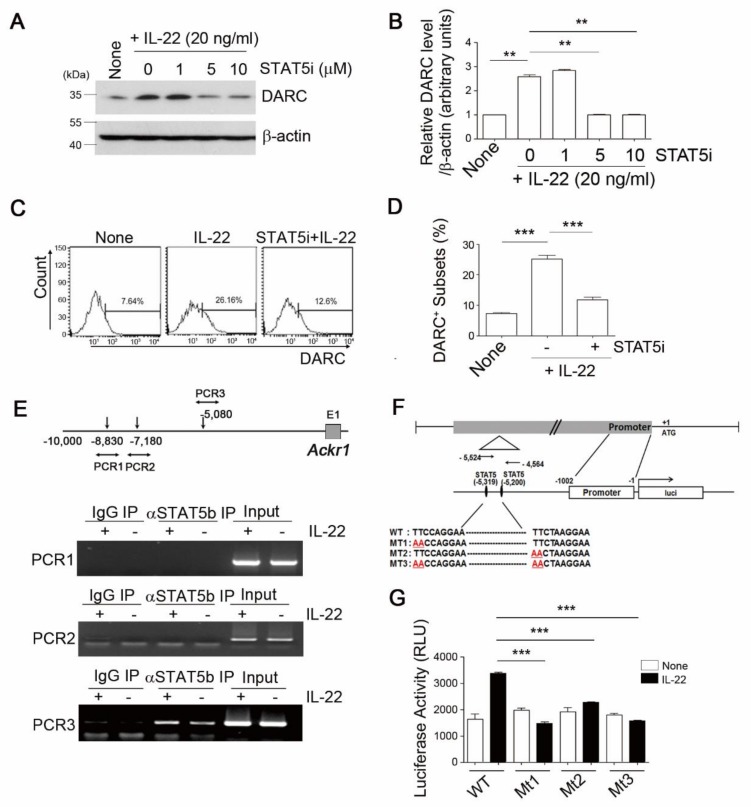
DARC expression in monocytes is dependent on an IL-22/IL-22Ra1-STAT5b signaling axis. (**A**–**D**) THP1 cells were incubated with 20 ng/mL IL-22 in the absence or presence of the indicated concentrations of the STAT5b inhibitor, CAS285989, for 24 h and DARC protein expression was determined by immunoblotting assay (**A**, **B**) and FACS analysis (**C**, **D**). Western blot was quantified and expressed as the ratio of DARC and ß-actin intensity. Data are representative of three independent experiments with similar results. (**E**) RT-PCR ChiP assay results showing the occupancy of p-STAT5 over the atypical chemokine receptor 1 (Ackr1) promoter. Three predicted STAT5b binding regions were located downstream from the core promoter region of *ackr1* (−10,000 to −4500) (**E**, upper). Interactions between STAT5b and the *ackr1* (DARC) promoter sequence with or without IL-22 treatment in THP-1 cells were determined by ChiP assay using specific PCR primers (1~3) (**E**, lower). (**F**) Firefly luciferase constructs containing the core promoter region of *ackr1* (−1001 to −1), PCR 3 primer-selective fragment (−5524 to −4564), and luciferase coding region in pGL3 enhancer vector. +1 indicates the transcriptional start site. (**G**) HEK 293 cells were co-transfected with the firefly luciferase constructs (WT, MT1, MT2, or MT3 of STAT5b binding sites), incubated for 24 h, and then treated with 20 ng/mL IL-22. A Renilla luciferase control was included in the dual-luciferase assay. The relative luciferase activity represents the dual luciferase activity ratio (firefly/Renilla luciferase). The results represent the mean ± SEM of at least three independent experiments. Comparisons were performed using ANOVA (multiple groups). ** *p* < 0.01, *** *p* < 0.001 corresponding to control group versus WT control.

**Figure 4:**
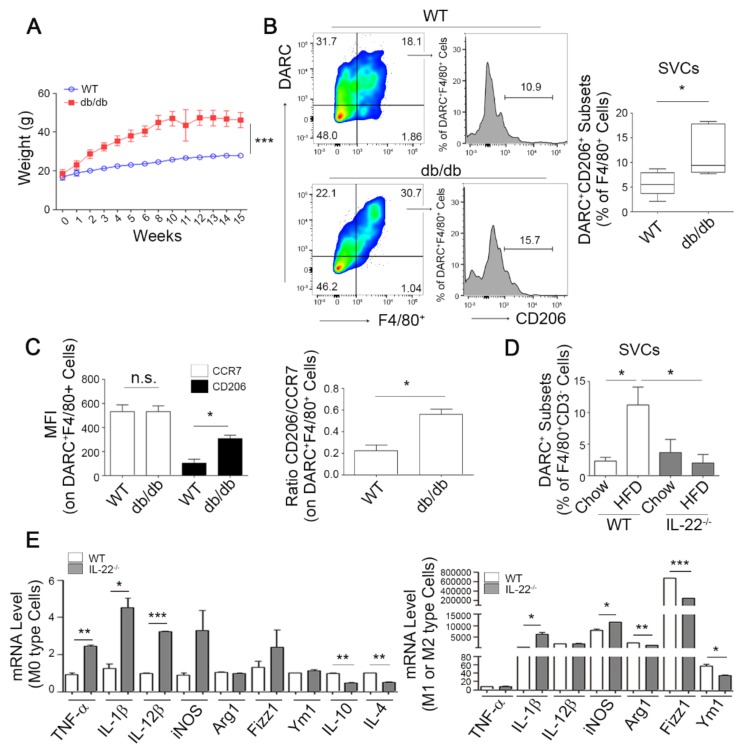
DARC^+^ macrophages show regulatory M2 characteristics under obese conditions. (**A**) Body weights of WT and db/db mice were monitored (0–15 weeks, *n* = 8 mice per group). (**B**) Frequency of CD206^+^DARC^+^ subsets in the monocyte/macrophage populations in SVCs from eWATs of WT and db/db mice. FACS plots identified CD206^+^DARC^+^ subsets in populations of myeloid cells with F4/80^+^ marker and the graph (right) shows the proportions of CD206^+^DARC^+^ in monocytes/macrophages of SVCs defined by F4/80^+^. (**C**) MFI of DARC^+^F4/80^+^ cells and ratio of CD206/CCR7 in DARC^+^F4/80^+^ cells were analyzed by FACS in WT and db/db mice. (**D**) The DARC^+^ subset is enhanced by an HFD corresponding to an increase in the Th22^+^ subset. Distribution of the DARC^+^ cells was analyzed among the SVCs of eWATs of *WT* and *IL-22*^−/−^mice subjected to a chow diet or an HFD. * *p* < 0.05 versus the corresponding control. (**E**) An IL-22 deficiency skews the monocytes/macrophages toward an inflammatory state. BMMs from WT and IL-22 KO mice were stimulated with either 25 ng/mL IFN-γ and 100 ng/mL LPS or 10 ng/mL IL-4 to derive M1 or M2 macrophages, respectively. qPCR was used to analyze the mRNA levels of TNF-α, IL-1β, IL-12β, and iNOS (M1 markers) and Arg1, Fizz1, Ym1, IL-4, and IL-10 (M2 markers) before (**E**, left) and after (**E**, right) stimulation. All samples were normalized to the GAPDH expression level. The results represent the mean ± SEM of at least three independent experiments. Comparisons were performed using Tukey post hoc comparison test. * *p* < 0.05, ** *p* < 0.01, *** *p* < 0.001 versus the corresponding control.

**Figure 5:**
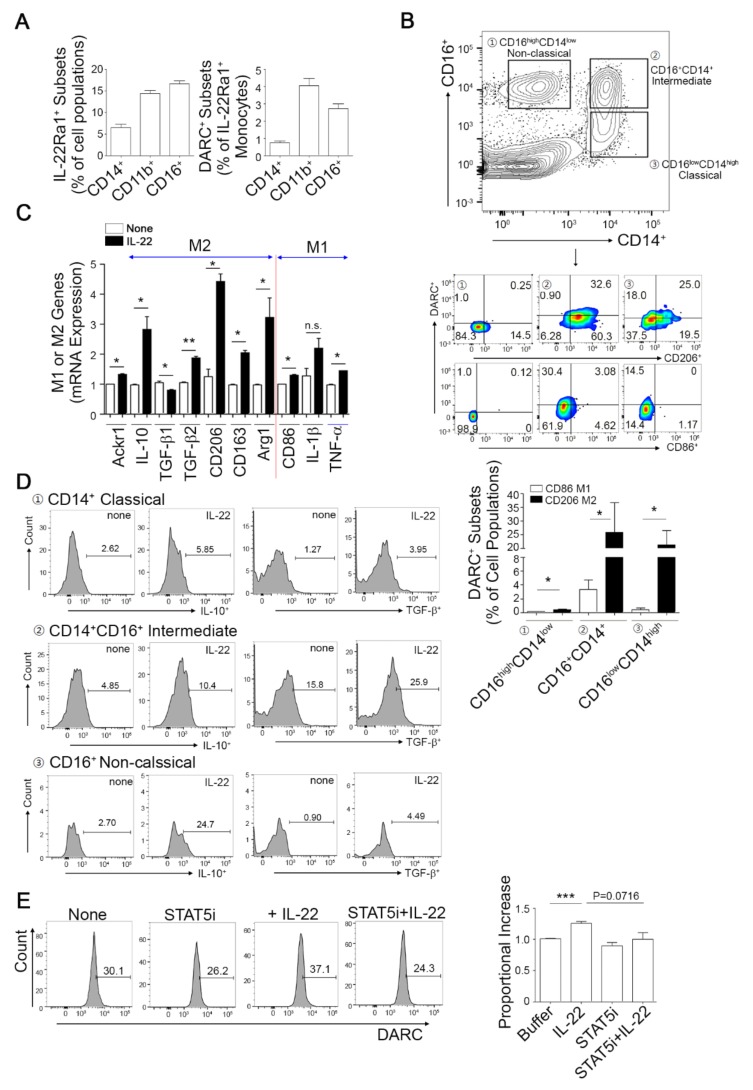
Presence of DARC^+^IL-22R1 cells in human peripheral blood. (**A**) The presence of a DARC^+^ subset among the monocytes or macrophages of human blood. The relative proportions of IL-22Ra1^+^- or DARC^+^- subset in monocytes/macrophages, defined by CD14^+^, CD11b^+^, or CD16^+^, were estimated in human PBMCs. (**B**) DARC^+^ cells are present among CD16^+^CD14^+^ macrophages. Myeloid monocyte subsets of human blood were gated as (1) CD16^hi^CD14^lo^-non-classical, (2) CD16^+^CD14^+^-intermediate, and (2) CD16^lo^CD14^hi^-classical monocytes (**B**, upper). The DARC^+^ subset frequency in cell subpopulations sorted using an inflammatory M1 maker, CD86^+^, or alternative M2 marker, CD206^+^, was quantified from selective populations of (1), (2) and (3) (**C**, middle and lower) using FACS analysis. * *p* < 0.05 versus the corresponding control. (**C**) IL-22 signaling enhances M2-like macrophage potential. PBMCs isolated from human healthy donors (*n* = 3~4) were treated with 20 ng/mL rIL-22 for 24 h and then classical inflammatory M1- or alternative M2-related genes were identified by qPCR. * *p* < 0.05, ** *p* < 0.01 versus the corresponding control. Values are expressed as a mean ± SEM. Comparisons were performed using *t*-tests (two groups). (**D**) Intracellular FACS analysis of cytokine-producing monocytes following IL-22 stimuli. Isolated PBMCs were stimulated with IL-22, incubated in the presence of brefeldin A, and then analyzed by FACS. Representative flow cytometry histograms of IL-10- and TGF-β-producing monocytes from gated DARC^+^ monocytes in blood are shown (healthy donors, *n* = 4). (**E**) STAT5 signaling is necessary for DARC expression via IL-22 in human primary monocytes. Flow cytometry was used to analyze DARC expression in peripheral blood mononuclear cells (PBMCs) isolated from three donors. Non-stimulated cells as resting PBMCs served as a control. Cells were pretreated with STAT5i for 30 min and incubated with or without 20 ng/mL of IL-22 for 24 h, followed by DARC expression analysis by FACS. *** *p* < 0.001 versus the corresponding control. Data are the mean ± SEM. Comparisons were performed using *t*-tests (two groups) or ANOVA (multiple groups). Data were combined from at least three independent experiments.

All quantitative experiments were performed at least three or more times. The results are expressed as a mean ± SEM or mean ± SD. ANOVA, student’s t test, post hoc, or Kruskal-Wallis tests were performed using GraphPad Prism VI software (GraphPad Software, Inc.). Tukey post hoc was performed for multiple to one comparison, as shown in Figure 4C and Figure S4. For two group mean comparison performed, as shown in Figures 1–5, and ANOVA test and Kruskal-Wallis tests (Figures 3–5) were used to compare the individual groups. *p* < 0.05, *p* < 0.01, or *p* < 0.001 was considered statistically significant.

## 3. Results

### 3.1. Increased Infiltration by a Distinct DARC^+^ Monocyte/Macrophage Subset of the Epididymal White Adipose Tissues of HFD-Fed Mice

It has been recently reported that CCL2 and CCL5 are linked to the obesity-associated infiltration by monocytes/macrophages of adipose tissues [34]. To determine the association between the chemokine gene expression signature and high fat diet-induced obesity, we analyzed the mRNA expression levels of obesity inflammation-related genes by extracting the publicly available Gene Expression Omnibus (GEO, GSE39549) dataset of epididymal white adipose tissues (eWATs) from normal diet (chow)-fed or HFD-fed mice [35]. We found that the CCL2, Ackr1 (DARC), IL-1β, and IL-10 transcript levels were enhanced in eWATs from the HFD-fed mouse groups (Figure S1). We also confirmed that CCL2 and CCL5 expression were prominently elevated in eWATs from the HFD-fed mice group, as analyzed by qPCR (Figure 1A).

Although DARC is regulated by a broad range of inflammatory chemokines, the expression and function of this receptor in myeloid cells has not been yet defined in detail. Recently, it has been shown that a DARC deficiency augments adipose tissue inflammation, accompanied by increased CCL2 and abundant macrophage CLS levels in adipose tissue [26]. Given the known involvement of DARC in adipose tissue inflammation [26] and our current observation of an increased expression of CCL2 and DARC in HFD-fed mice (Figure S1, Figure 1A), we postulated that a subset of myeloid cells in adipose tissue may express DARC. We subsequently found that myeloid cells as well as CD4^+^, CD8^+^, and B cells (data not shown) express DARC in the stromal vascular cells (SVCs) from the eWATs of wild-type (WT) mice (Figure 1B). In this experiment, DARC^+^CD11b^+^ and DARC^+^F4/80^+^ cells constituted 6.64 ± 0.51 % and 18.1 ± 0.71 % of the total myeloid cell population, respectively (Figure 1B, right), suggesting that these cells have macrophage characteristics in adipose tissue. In comparison, a smaller population of the cells positive for granulocyte-associated marker Gr-1 expressed DARC (0.29 ± 1.34%, Figure 1B). DARC was predominantly expressed in a CD11b^hi^F4/80^hi^ subset of myeloid monocytes as well as in CD11b^lo^F4/80^hi^ fraction of adipose resident macrophages, but in only 2.55 ± 0.13% of CD11b^hi^F4/80^lo^ myeloid monocytes (Figure 1C). We thus identified a previously unknown subset of monocyte-derived DARC^+^ macrophages in epididymal fat adipose tissues.

Obesity that is characterized by chronic low-grade systemic inflammation is associated with an elevated level of CCL2 and CCL5 [17,18], which are the major chemokines involved in macrophage infiltration and the alteration of decoy receptor expression [36,37,38]. We therefore examined whether an HFD would induce the infiltration of eWAT tissue by DARC^+^ cells. Our analysis indicated that the DARC protein was abundantly expressed in islet or crown like immune cell clusters in the adipose tissue from HFD-fed mice (Figure 1D). Moreover, F4/80^+^ macrophages and caveolin were substantially positively stained in these mice and also co-stained with DARC (Figure S3). Interestingly, the DARC^+^ cell subset was markedly increased in Ly6C^lo^ cells (anti-inflammatory) but not Ly6C^hi^ cells (inflammatory) within the CD11b^+^CD3^−^ monocyte pool in the BM and spleen after HFD administration (Figure 1E,F). In addition, we noted that the DARC^+^ fraction among CD11b^+^F4/80^+^ monocytes was 2.1- (*p* < 0.01) and 3.5- (*p* < 0.001) fold higher in the peritoneum and among the SVCs, respectively, from the HFD mice (Figure 1G,H). These results indicate that obese stress may induce the infiltration of adipose tissue by a DARC^+^ monocyte subset.

### 3.2. IL-22 Enhances DARC Expression in CD11b^+^ or F4/80^+^ Myeloid Monocytes/Macrophages

Previous studies have suggested that diet-induced obesity leads to an ATM shift from an alternative M2-polarized state to a classical M1 proinflammatory state [10,13]. To define the polarization status of DARC^+^ subset monocytes/macrophages in adipose tissue, we used CD11b^+^F4/80^+^CCR7^+^ and CD11b^+^F4/80^+^CD206^+^ cells as markers of M1 and M2 macrophages, respectively [39]. Surprisingly, the frequency of DARC^+^ cells in alternative M2 macrophages of the CD206^+^ subset was much greater than that in classical M1 macrophages in adipose tissue (Figure 2A,B). This CD206^+^DARC^+^ subset, but not the CCR7^+^DARC^+^ subset, of CD11b^+^F4/80^+^ cells was significantly elevated in adipose tissue from HFD-fed mice fed (Figure 2C,D), implying that DARC^+^ macrophages display M2-like characteristics.

Flow cytometry analysis further revealed that 13.9 ± 2.65% and 3.35 ± 0.54% of Ly6C^lo^CD11b^+^ and Ly6C^hi^CD11b^+^ cell population in the spleen expressed DARC, respectively (*p* < 0.001, Figure 2E). This was accompanied by an increased number of cells positive for IL-22Ra1, a cognate receptor of IL-22 (Figure 2E). The Ly6C^hi^CD11b^+^ population expressed lower levels of DARC and IL-22Ra1 (Figure 2E), raising the possibility that IL-22 signaling may modulate DARC expression. We explored the possibility of an IL-22-dependent induction of DARC expression in BM cells. As shown in Figure 2F, BM cells treated with IL-22 yielded a substantially increased DARC^+^ monocyte subpopulation from the CD11b^+^ subset (*p* < 0.001). Accordingly, an enhanced population of F4/80^+^ myeloid monocytes was observed in parallel following IL-22 exposure in a similar fashion (*p* < 0.0001, Figure 2F). This suggested that IL-22 serves as an important determinant of DARC induction in CD11b^+^ or F4/80^+^ myeloid monocytes/macrophages.

### 3.3. STAT5 is an Essential Mediator of IL-22-Induced DARC Expression in Monocytes

The IL-22 induction of the DARC protein levels in THP-1 cells was completely blocked by a STAT5 signal inhibitor (STAT5i), but not inhibitors of NF-κB, ERK, JNK, or p38 (data not shown). STAT5i exposure abolished the IL-22-dependent increase in DARC in a concentration-dependent manner, as evidenced by immunoblotting (Figure 3A,B) and flow cytometry (Figure 3C,D).

To elucidate the direct participation of STAT5 in “IL-22-DARC” signaling, we analyzed the distal 5-flanking sequence of the *Ackr1* gene and found three potential stat5b consensus binding sequences (Figure 3E, upper). DNA fragments immunoprecipitated with anti-phospho-STAT5b antibody were PCR-amplified with the −8830 F/R primer (PCR1), −7180 F/R primer (PCR2), or −5080 F/R primer (PCR3). A 267 bp amplicon was generated using the −5080 F/R primers (PCR3), but not with the other fractions. This binding of stat5b to the *Ackr1* gene was modestly increased in response to IL-22 (Figure 3E, lower). To further evaluate this potential stat5b response element, we constructed various *Ackr1* promoter constructs with a luciferase reporter containing wild type (WT) or mutated forms (MT) of the putative stat5 binding site: WT, MT1 (TTCC to AACC), MT2 (TTCT to AACT), and MT3 (TTCC to AACC and TTCT to AACT) (Figure 3F). Co-transfection assays with the WT form revealed that IL-22 treatment led to a two-fold increase in Ackr1 promoter activity in 293T cells (Figure 3G). However, mutation of either (MT1 and MT2) or both sites (MT3) of the stat5 promoter element completely abolished this effect (Figure 3G), indicating that these two predicted target sites are required for STAT5-dependent DARC expression. Taken together, these results suggest that the transcriptional factor STAT5 directly binds to distinct motifs in the Ackr1 promoter and thereafter induces DARC expression in monocytes.

### 3.4. DARC^+^ Macrophages Show Regulatory M2 Characteristics under Obese Conditions

To confirm the infiltration of DARC^+^ monocytes under obesity conditions, we compared the frequency of CCR7^+^DARC^+^ or CD206^+^DARC^+^ subsets in the monocyte/macrophage populations in SVCs between WT and db/db mice. With respect to obesity-related alteration, db/db mice exhibited a pronounced weight gain compared with WT mice (Figure 4A). Importantly, the percentage and mean fluorescence intensity (MFI) of DARC^+^CD206^+^ subset in F4/80^+^ cells of db/db mice was much higher than that of WT mice (Figure 4B,C). In comparison, MFI of DARC^+^CCR7^+^ subset displayed no significant difference between WT and db/db mice, yielding higher ratio of CD206/CCR7 in db/db mice (Figure 4C). These results suggest that DARC^+^ macrophages show regulatory M2 characteristics in obese conditions.

Furthermore, the accumulation of IL-22-producing T cells (Th22^+^ cells) from the CD4^+^CD3^+^ population in response to the HFD in WT mice was modestly increased in adipose tissue (*p* = 0.078, Figure S4), in agreement with a previous report [40]. However, the IL-22-depleted mice failed to increase the accumulation of these cells (Figure S4). Notably, HFD feeding significantly expanded the number of DARC^+^ monocytes within the F4/80^+^CD3^−^ population in SVCs from WT mice eWATs, while IL-22-depleted mice demonstrated no increase in the number of these cells (Figure 4D). Collectively, these results suggest that IL-22 may increase the infiltration of DARC^+^ monocytes in adipose tissue and thereby regulate fat inflammation responses associated with HFD feeding.

With respect to macrophage polarization, M2 macrophage-associated genes including IL-4 and TGF-β2 as well as DARC and IL-22Ra1, but not M1 polarization marker genes, were found to be significantly increased by IL-22 treatment in bone marrow-derived macrophages (BMMs) (Figure S5), indicating that IL-22 acts on macrophages to promote M2 polarization. Further qPCR analyses revealed that macrophages from IL-22-depleted mice expressed higher levels of M1 marker genes (e.g., TNF-α, IL-1β, and IL-12β) but lower expression of M2 maker genes (e.g., IL-10 and IL-4) even before differentiation (Figure 4E, left). After differentiation into M1 (LPS/IFN-γ stimulation) or M2 (IL-4 stimulation) macrophages, the expression of M1 markers such as IL-1β and iNOS was significantly increased in macrophages from IL-22-depleted mice, while the acquisition of alternative M2 marker was impaired (Figure 4E, right). This suggested that IL-22 depletion may skew BMM differentiation toward an inflammatory state. These results collectively suggest that IL-22 induces a shift toward alternative M2-like macrophage polarization.

### 3.5. Frequencies of DARC^+^ Mononuclear Cells in Human Peripheral Blood

We assessed whether DARC induction in monocyte/macrophages by IL-22 would also occur in human cells. We detected the presence of a DARC^+^ fraction in the monocytes or macrophages among human PBMCs (Figure S6, middle), which had a higher expression of IL-22R1 (Figure S6, lower). Previous studies have reported very low frequencies of IL-22R1-expressing cells from total PBMCs under physiological and some pathologic conditions [41,42]. Notably however, we here identified IL-22R1-expressing cells and/or DARC^+^IL-22R1^+^ monocytes in the CD14^+^, CD11b^+^, and CD16^+^ fraction in PBMCs from healthy individuals (Figure 5A). Because most CD14^+^ and/or CD16^+^ monocytes contain a CD11b^+^ subset, to more clearly deciphering DARC expressing mononuclear cell subset, myeloid monocytes were subdivided into three populations depending on the levels of CD14 and CD16 i.e., CD16^lo^CD14^hi^ classical, CD16^+^CD14^+^ intermediate, or CD16^hi^CD14^lo^ non-classical monocyte subset. We then monitored the expression levels of DARC, CD86 as an inflammatory M1 maker, and CD206 as an alternative M2 marker using fluorescence-activated cell sorting (FACS) (Figure 5B, upper and middle). The numbers of DARC^+^ cells were higher in the CD16^lo^CD206^+^CD14^+^ classical and CD16^hi^CD206^+^CD14^+^ intermediate subsets than in the CD16^hi^ CD206^+^CD14^lo^ non-classical monocyte group (Figure 5B, middle). CD206^+^ M2-like cells were found to express a higher level of DARC compared to CD86^+^ M1-like cells (Figure 5B, lower), suggesting that the CD14^+^ and/or CD16^+^ monocytes/macrophages among human PBMCs possess primitive DARC^+^IL-22Ra1^+^ cell subset that has an M2-like macrophage functional potential.

The expression of M1- or M2-related genes was evaluated in PBMCs treated with or without IL-22 to determine the impact of IL-22 stimuli on monocyte polarization. As shown in Figure 5C, DARC expression was enhanced by IL-22. Of note, the levels of M2-polarized genes including IL-10, TGF-β2, CD206, CD163, and Arg1 were predominantly increased by IL-22 stimulation compared to those of M1-polarized genes such as CD86 and TNF-α. In addition, the IL-10 and TGF-β expression levels following IL-22 exposure in CD14^+^ classical, CD14^+^CD16^+^ intermediate, or CD16^+^ non-classical monocyte DARC^+^ subsets were upregulated by 2.2-, 2.0-, or 9.14-fold for IL-10 and 3.11-, 1.6-, or 4.0-fold for TGF-β, respectively, compared to the non-treated control groups (Figure 5D). Flow cytometry analysis using human PBMCs also revealed that DARC expression was enhanced by IL-22 stimuli and that this response was modestly inhibited by STAT5i (Figure 5E), further demonstrating that STAT5 activation is a necessary prerequisite for the induction of DARC expression via an IL-22 signaling axis. These results suggest that IL-22 treatment increases IL-10 and TGF-β production, thereby causing monocytes to have M2-like potential.

## 4. Discussion

We have for the first time identified a discrete subset of epididymal fat adipose tissue-resident M2-like DARC^+^ macrophages expressing the IL-22 receptor. Our results show that IL-22 can regulate the DARC^+^ macrophage pool via a STAT5b signaling axis and that the loss of IL-22 reduced DARC^+^ macrophage subpopulation upon HDF consumption. This indicates that IL-22 is a critical component for the infiltration of DARC^+^ macrophages.

As IL-22-secreting cells are highly prevalent in the GI tract, where nutrients are absorbed, it is likely that a cross-talk between these cells and myeloid cells is involved in the adipose tissue generation balance. The cumulative evidence to date suggests that IL-22 plays a role in the immune-inflammatory system in context of obesity and its metabolic consequences, but that the role of IL-22 in metabolic regulation is extremely complex and thus still controversial [27,43,44,45]. It has been reported that IL-22 does not play any role in the development of adiposity and metabolic alterations in mice [28,46]. However, it was intriguing to note that IL-22 markedly reduces body weight, decreases blood glucose levels, and alleviates the metabolic complications induced by HFD [28,43,44]. In contrast, higher levels of IL-22-producing Th cells were observed in humans with type 2 diabetes mellitus (DM2) [47] and blood IL-22 levels in obese individuals were found to be elevated in DM2 patients compared to control subjects [31]. Furthermore, the number of IL-22^+^CD4^+^ cells was shown to be higher in the adipose tissue of obese subjects [31,32]. Although the precise generation mechanism and functional differences in DARC expression between RBCs and WBCs were not clearly explained by our analysis, considering the chemokine sinking or trap function of DARC in RBCs, we speculate that the elevated chemokine and inflammatory responses might be counterbalanced by the increased DARC^+^ mononuclear cell numbers.

One of interesting findings from our current study is that the DARC^+^ cells express IL-22R1, which is mainly located in peripheral tissues such as the skin and gut mucosa. Surprisingly, over 30% of the DARC^+^ cells exhibited IL-22R1 expression, which correlated well with their generation (Figure 2). IL-22 is a tightly regulated pro- and anti-inflammatory member of the IL-10 family that signals through the IL-22 receptors, and is composed of a ubiquitous IL-10R2 subunit and a restrictively expressed IL-22R1 subunit [29,48]. Even though the primary role of immune cells, especially Th cells, is the production of IL-22, it is well-known that several immune cells can induce IL-22R1 under certain pathologic conditions such as mycobacterium infected macrophages [49,50] and leukemia [51]. Hence, it is perhaps unsurprising to find that IL-22R1 is expressed in certain mononuclear cell population in obese conditions.

DARC^+^Ly6C^lo^ ATMs are found to express high levels of CCR2-like M1 type macrophages. However, besides the CCR2 level, DARC^+^Ly6C^lo^ ATMs express a higher level of IL-10, IDO, and TGF-β mRNA than those of DARC^-^ cells, indicating that the DARC^+^Ly6C^lo^ cells essentially function to revert the inflammatory status in diet-induced obesity to an anti-inflammatory status. We found increased levels of IL-22-secreting T cells in the lamina propria of the small intestine after administration of an HFD (data not shown). Taken together, these results suggest that DARC^+^Ly6C^lo^ ATMs induced by IL-22 under HFD conditions may have anti-inflammatory properties, whereby they reduce immune cell infiltration and suppress inflammatory cytokine production.

There were a few limitations of this study that must be considered when interpreting its findings. As we focused on the role of IL-22 for infiltration of DARC^+^Ly6C^lo^ ATMs, the functional characteristics of these cells were not assessed using DARC KO mice or a neutralizing model. The distribution of DARC^+^Ly6C^lo^ cells in the body, (e.g., liver, visceral lymph nodes, spleen) and functional role of these cells in obesity-related pathologic conditions needs to be determined. Lastly, as we focused on IL-22 and DARC^+^ cell generation, future experiments to define the functional role of these cells in obesity or adipogenesis should be conducted with respect to the differences in surface marker levels (e.g., CD16^hi^DARC^+^ vs. CD16^Int^DARC^+^), ontogeny, developmental changes in DARC^+^ cells, and changes in the Treg cell levels in adipose tissue. Of the three allele variants of DARC in human populations, Fy glycoprotein*A (FY*A) and FY*B are common in Asia and Europe, respectively. In comparison, FY*O allele has been shown to protect against malarial parasites, Plasmodium vivax in sub-Saharan Africa. The frequencies of FY*A antigens of human blood cell in our study with Asian human subjects was 91% and we detected the presence of DARC^+^IL-22R1 cells in human peripheral blood. The presence of these cells in other populations should be determined.

In conclusion, we have for the first time identified a distinct subset of DARC^+^Ly6C^lo^ ATMs expressing IL-22R1 within adipose tissue as a novel myeloid cell type induced by IL-22. This may indicate the presence of a rare population of IL-22-responsive myeloid cells which interact with IL-22 -secreting T cells in a paracrine manner. To clearly elucidate the functional role of DARC^+^Ly6C^lo^ ATMs in obesity, experiments using DARC KO mice or other neutralizing models should be performed in the future. In addition to those in fat tissues, the roles of DARC^+^Ly6C^lo^ myeloid cells in atherosclerosis, fatty changes in the liver, and other obesity-related pathologic lesions should be investigated.

## Supplementary Materials

The following are available online at https://www.mdpi.com/2073-4409/8/12/1587/s1. Figure S1: Elevated expression of Ackr1, ccl2, Il-1β, and IL10 transcripts in epididymal fat adipose tissue (eWAT) from high fat diet (HFD)-induced obese mice. Figure S2: Histogram strategy of isotype control for CD11b-F4/80^+^, CD11b^+^F4/80^+^, or CD11b^+^F4/80^lo^. Figure S3: DARC, F4/80, and caveolin expression was determined using immunofluorescent staining. Figure S4: Distribution of the Th22^+^ subset in eWAT SVCs after HFD consumption. Figure S5. The IL-22 signaling axis enhances M2-like monocyte-derived macrophage potential in the mouse. Figure S6. Identification of DARC^+^ monocytes in human PBMCs. Table S1: PCR primers used in this study.

## Abbreviations

Adipose tissue macrophages (ATMs), atypical chemokine receptor 1 (ACKR1), crown-like structures (CLSs), 4′,6-diamidine-2′-phenylindole dihydrochloride (DAPI). Duffy antigen receptor for chemokine (DARC), epididymal white adipose tissues (eWATs), fluorescence-activated cell sorting (FACS), Hank’s balanced salt solution (HBSS), high-fat diet (HFD), interleukin 22 (IL-22), knockout (KO), peripheral blood mononuclear cells (PBMCs), stromal vascular fraction cells (SVCs), phosphate-buffered saline (PBS).

## References

[1] Galic S., Oakhill J.S., Steinberg G.R. (2010). Adipose tissue as an endocrine organ. Mol. Cell Endocrinol..

[2] Vieira-Potter V.J. (2014). Inflammation and macrophage modulation in adipose tissues. Cell Microbiol..

[3] Francisco V., Pino J., Campos-Cabaleiro V., Ruiz-Fernandez C., Mera A., Gonzalez-Gay M.A., Gomez R., Gualillo O. (2018). Obesity, Fat Mass and Immune System: Role for Leptin. Front. Physiol..

[4] Stefanovic-Racic M., Yang X., Turner M.S., Mantell B.S., Stolz D.B., Sumpter T.L., Sipula I.J., Dedousis N., Scott D.K., Morel P.A. (2012). Dendritic cells promote macrophage infiltration and comprise a substantial proportion of obesity-associated increases in CD11c+ cells in adipose tissue and liver. Diabetes.

[5] Winer D.A., Winer S., Shen L., Wadia P.P., Yantha J., Paltser G., Tsui H., Wu P., Davidson M.G., Alonso M.N. (2011). B cells promote insulin resistance through modulation of T cells and production of pathogenic IgG antibodies. Nat. Med..

[6] Foryst-Ludwig A., Hartge M., Clemenz M., Sprang C., Hess K., Marx N., Unger T., Kintscher U. (2010). PPARgamma activation attenuates T-lymphocyte-dependent inflammation of adipose tissue and development of insulin resistance in obese mice. Cardiovasc. Diabetol..

[7] Kintscher U., Hartge M., Hess K., Foryst-Ludwig A., Clemenz M., Wabitsch M., Fischer-Posovszky P., Barth T.F., Dragun D., Skurk T. (2008). T-lymphocyte infiltration in visceral adipose tissue: A primary event in adipose tissue inflammation and the development of obesity-mediated insulin resistance. Arterioscler. Thromb. Vasc. Biol..

[8] Boutens L., Stienstra R. (2016). Adipose tissue macrophages: Going off track during obesity. Diabetologia.

[9] Weisberg S.P., McCann D., Desai M., Rosenbaum M., Leibel R.L., Ferrante A.W. (2003). Obesity is associated with macrophage accumulation in adipose tissue. J. Clin. Invest..

[10] Lumeng C.N., Bodzin J.L., Saltiel A.R. (2007). Obesity induces a phenotypic switch in adipose tissue macrophage polarization. J. Clin. Invest..

[11] Lumeng C.N., DelProposto J.B., Westcott D.J., Saltiel A.R. (2008). Phenotypic switching of adipose tissue macrophages with obesity is generated by spatiotemporal differences in macrophage subtypes. Diabetes.

[12] Gericke M., Weyer U., Braune J., Bechmann I., Eilers J. (2015). A method for long-term live imaging of tissue macrophages in adipose tissue explants. Am. J. Physiol. Endocrinol. Metab..

[13] Kawanishi N., Yano H., Yokogawa Y., Suzuki K. (2010). Exercise training inhibits inflammation in adipose tissue via both suppression of macrophage infiltration and acceleration of phenotypic switching from M1 to M2 macrophages in high-fat-diet-induced obese mice. Exercise Immunol. Rev..

[14] Thomas D., Apovian C. (2017). Macrophage functions in lean and obese adipose tissue. Metabolism.

[15] Cautivo K.M., Molofsky A.B. (2016). Regulation of metabolic health and adipose tissue function by group 2 innate lymphoid cells. Eur. J. Immunol..

[16] Bai Y., Sun Q. (2015). Macrophage recruitment in obese adipose tissue. Obes. Rev..

[17] Yao L., Herlea-Pana O., Heuser-Baker J., Chen Y., Barlic-Dicen J. (2014). Roles of the chemokine system in development of obesity, insulin resistance, and cardiovascular disease. J. Immunol. Res..

[18] Ota T. (2013). Chemokine systems link obesity to insulin resistance. Diabetes Metab. J..

[19] Kanda H., Tateya S., Tamori Y., Kotani K., Hiasa K., Kitazawa R., Kitazawa S., Miyachi H., Maeda S., Egashira K. (2006). MCP-1 contributes to macrophage infiltration into adipose tissue, insulin resistance, and hepatic steatosis in obesity. J. Clin. Invest..

[20] Kamei N., Tobe K., Suzuki R., Ohsugi M., Watanabe T., Kubota N., Ohtsuka-Kowatari N., Kumagai K., Sakamoto K., Kobayashi M. (2006). Overexpression of monocyte chemoattractant protein-1 in adipose tissues causes macrophage recruitment and insulin resistance. J. Biol. Chem..

[21] Hansell C.A., Hurson C.E., Nibbs R.J. (2011). DARC and D6: Silent partners in chemokine regulation?. Immunol. Cell Biol..

[22] Xu L., Ashkenazi A., Chaudhuri A. (2007). Duffy antigen/receptor for chemokines (DARC) attenuates angiogenesis by causing senescence in endothelial cells. Angiogenesis.

[23] De Brevern A.G., Wong H., Tournamille C., Colin Y., Le Van Kim C., Etchebest C. (2005). A structural model of a seven-transmembrane helix receptor: The Duffy antigen/receptor for chemokine (DARC). Biochim. Biophys. Acta.

[24] Nibbs R.J., Graham G.J. (2013). Immune regulation by atypical chemokine receptors. Nat. Rev. Immunol..

[25] Pruenster M., Mudde L., Bombosi P., Dimitrova S., Zsak M., Middleton J., Richmond A., Graham G.J., Segerer S., Nibbs R.J. (2009). The Duffy antigen receptor for chemokines transports chemokines and supports their promigratory activity. Nat. Immunol..

[26] Benson T.W., Weintraub D.S., Crowe M., Yiew N.K.H., Popoola O., Pillai A., Joseph J., Archer K., Greenway C., Chatterjee T.K. (2018). Deletion of the Duffy antigen receptor for chemokines (DARC) promotes insulin resistance and adipose tissue inflammation during high fat feeding. Mol. Cell Endocrinol..

[27] Sabat R., Wolk K. (2015). Deciphering the role of interleukin-22 in metabolic alterations. Cell Biosci..

[28] Wang X., Ota N., Manzanillo P., Kates L., Zavala-Solorio J., Eidenschenk C., Zhang J., Lesch J., Lee W.P., Ross J. (2014). Interleukin-22 alleviates metabolic disorders and restores mucosal immunity in diabetes. Nature.

[29] Sargent J. (2014). Immunology: IL-22 and metabolic disease. Nat. Rev. Endocrinol..

[30] Sonnenberg G.F., Fouser L.A., Artis D. (2011). Border patrol: Regulation of immunity, inflammation and tissue homeostasis at barrier surfaces by IL-22. Nat. Immunol..

[31] Fabbrini E., Cella M., McCartney S.A., Fuchs A., Abumrad N.A., Pietka T.A., Chen Z., Finck B.N., Han D.H., Magkos F. (2013). Association between specific adipose tissue CD4+ T-cell populations and insulin resistance in obese individuals. Gastroenterology.

[32] Dalmas E., Venteclef N., Caer C., Poitou C., Cremer I., Aron-Wisnewsky J., Lacroix-Desmazes S., Bayry J., Kaveri S.V., Clement K. (2014). T cell-derived IL-22 amplifies IL-1beta-driven inflammation in human adipose tissue: Relevance to obesity and type 2 diabetes. Diabetes.

[33] Liang G., Lin J.C., Wei V., Yoo C., Cheng J.C., Nguyen C.T., Weisenberger D.J., Egger G., Takai D., Gonzales F.A. (2004). Distinct localization of histone H3 acetylation and H3-K4 methylation to the transcription start sites in the human genome. Proc. Natl. Acad. Sci. USA.

[34] Xue W., Fan Z., Li L., Lu J., Zhai Y., Zhao J. (2019). The chemokine system and its role in obesity. J. Cell. Physiol..

[35] Kwon E.Y., Shin S.K., Cho Y.Y., Jung U.J., Kim E., Park T., Park J.H., Yun J.W., McGregor R.A., Park Y.B. (2012). Time-course microarrays reveal early activation of the immune transcriptome and adipokine dysregulation leads to fibrosis in visceral adipose depots during diet-induced obesity. BMC Genom..

[36] Stone M.J., Hayward J.A., Huang C., Z E.H., Sanchez J. (2017). Mechanisms of Regulation of the Chemokine-Receptor Network. Int. J. Mol. Sci..

[37] Di Liberto D., Locati M., Caccamo N., Vecchi A., Meraviglia S., Salerno A., Sireci G., Nebuloni M., Caceres N., Cardona P.J. (2008). Role of the chemokine decoy receptor D6 in balancing inflammation, immune activation, and antimicrobial resistance in Mycobacterium tuberculosis infection. J. Exp. Med..

[38] Hughes C.E., Nibbs R.J.B. (2018). A guide to chemokines and their receptors. FEBS J..

[39] Shapouri-Moghaddam A., Mohammadian S., Vazini H., Taghadosi M., Esmaeili S.A., Mardani F., Seifi B., Mohammadi A., Afshari J.T., Sahebkar A. (2018). Macrophage plasticity, polarization, and function in health and disease. J. Cell. Physiol..

[40] Dudakov J.A., Hanash A.M., van den Brink M.R. (2015). Interleukin-22: Immunobiology and pathology. Annu. Rev. Immunol..

[41] Donnelly R.P., Sheikh F., Kotenko S.V., Dickensheets H. (2004). The expanded family of class II cytokines that share the IL-10 receptor-2 (IL-10R2) chain. J. Leukocyte Biol..

[42] Lecart S., Morel F., Noraz N., Pene J., Garcia M., Boniface K., Lecron J.C., Yssel H. (2002). IL-22, in contrast to IL-10, does not induce Ig production, due to absence of a functional IL-22 receptor on activated human B cells. Int. Immunol..

[43] Liang S.C., Nickerson-Nutter C., Pittman D.D., Carrier Y., Goodwin D.G., Shields K.M., Lambert A.J., Schelling S.H., Medley Q.G., Ma H.L. (2010). IL-22 induces an acute-phase response. J. Immunol..

[44] Hasnain S.Z., Borg D.J., Harcourt B.E., Tong H., Sheng Y.H., Ng C.P., Das I., Wang R., Chen A.C., Loudovaris T. (2014). Glycemic control in diabetes is restored by therapeutic manipulation of cytokines that regulate beta cell stress. Nat. Med..

[45] Yang L., Zhang Y., Wang L., Fan F., Zhu L., Li Z., Ruan X., Huang H., Wang Z., Huang Z. (2010). Amelioration of high fat diet induced liver lipogenesis and hepatic steatosis by interleukin-22. J. Hepatol..

[46] Park O., Ki S.H., Xu M., Wang H., Feng D., Tam J., Osei-Hyiaman D., Kunos G., Gao B. (2015). Biologically active, high levels of interleukin-22 inhibit hepatic gluconeogenesis but do not affect obesity and its metabolic consequences. Cell Biosci..

[47] Guo H., Xu B.C., Yang X.G., Peng D., Wang Y., Liu X.B., Cui C.R., Jiang Y.F. (2016). A High Frequency of Peripheral Blood IL-22(+) CD4(+) T Cells in Patients With New Onset Type 2 Diabetes Mellitus. J. Clin. Lab. Anal..

[48] Rutz S., Wang X., Ouyang W. (2014). The IL-20 subfamily of cytokines—From host defence to tissue homeostasis. Nat. Rev. Immunol..

[49] Treerat P., Prince O., Cruz-Lagunas A., Munoz-Torrico M., Salazar-Lezama M.A., Selman M., Fallert-Junecko B., Reinhardt T.A., Alcorn J.F., Kaushal D. (2017). Novel role for IL-22 in protection during chronic Mycobacterium tuberculosis HN878 infection. Mucosal. Immunol..

[50] Zeng G., Chen C.Y., Huang D., Yao S., Wang R.C., Chen Z.W. (2011). Membrane-bound IL-22 after de novo production in tuberculosis and anti-Mycobacterium tuberculosis effector function of IL-22^+^ CD4^+^ T cells. J. Immunol..

[51] Bard J.D., Gelebart P., Anand M., Amin H.M., Lai R. (2008). Aberrant expression of IL-22 receptor 1 and autocrine IL-22 stimulation contribute to tumorigenicity in ALK^+^ anaplastic large cell lymphoma. Leukemia.

