# Supplementary material for: Interleukin-22 Induces the Infiltration of Visceral Fat Tissue by a Discrete Subset of Duffy Antigen Receptor for Chemokine-Positive M2-Like Macrophages in Response to a High Fat Diet
Source: Cells. 2019 Dec 6;8(12):1587. doi: 10.3390/cells8121587 (PMC6953014; doi:10.3390/cells8121587)
Supplement: Supplementary file 1 [file cells-08-01587-s001.pdf]

Supplementary Materials

Methods

Intracellular staining and FACS analysis

For intracellular staining, human PBMCs were stimulated for 24 h with IL-22 and additionally incubated for 6 h at 37 °C with GolgiStop (BD). Stimulated cells were washed with PBS and stained with Fixable Viability Dye eFluor450 (eBioscience) to gate the live cells. Cells were then fixed and permeabilized using the commercially available intracellular Cytofix/Cytoperm kit (BD) as per the manufacturer's protocol. Antibodies used for surface and intracellular staining included: CD14-PerCP/cy5.5, CD16-FITC, IL-10-PE-Cy7, or TGF-β-PE (from BioLegends), and DARC-APC (R&D Systems). All data were acquired on an BD FACSCanto II (BD Biosciences) and analyzed using the FlowJo software (Tree Star, Ashland, OR).

Immunofluorescence staining analysis

Formalin-fixed and paraffin-embedded adipose tissues or spleen sections were also used for immunofluorescent staining. The tissues were blocked with 1% BSA in PBS for 1 h and incubated with their respective primary antibodies for 2 h. After washing, the cells were stained with 4',6-diamidine 2'-phenylindole dihydrochloride (DAPI, Invitrogen) or dye conjugated Alexa Fluor 488 (for DARC) or Alexar Fluor 649 (for caveolin or F4/80) secondary antibodies. Sections were then mounted, and DARC and caveolin or F4/80 localization was detected using a confocal laser scanning microscope (LSM 710; Carl Zeiss, Tokyo, Japan).

Supplementary Figure Legends

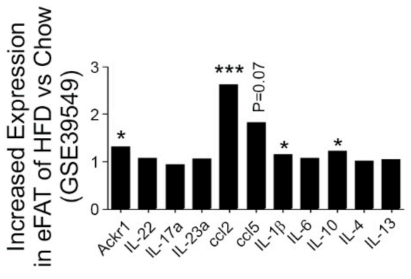

Figure S1

Figure S1. Elevated expression of Ackr1, ccl2, Il-1β, and IL10 transcripts in epididymal fat adipose tissue (eWAT) from high fat diet (HFD)-induced obese mice**.** Expression data were extracted from the Gene Expression Omnibus (GEO) dataset GSE39549. Data are the means ± SD of three independent experiments. **p* < 0.05, ****p* < 0.001 versus chow.

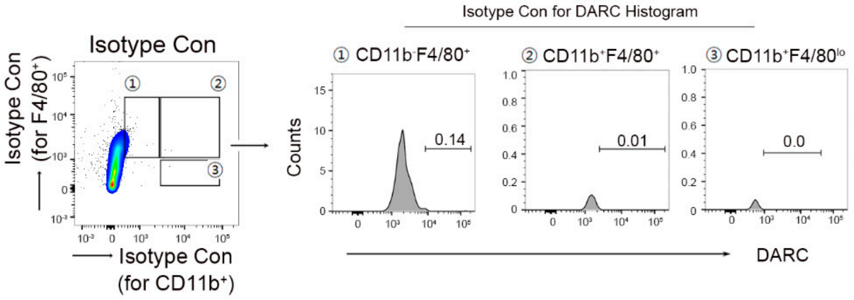

Figure S2

Figure S2. Histogram strategy of isotype control for CD11b-F4/80+, CD11b+F4/80+, or CD11b+F4/80lo. Shaded histograms represent isotype control corresponding to CD11b-F4/80+, CD11b+F4/80+, or CD11b+F4/80lo each. DARC subsets on CD11b-F4/80+, CD11b+F4/80+, or CD11b+F4/80lo from SVCs were determined by flow cytometry in Figure 1C. Data are representative of three independent experiments.

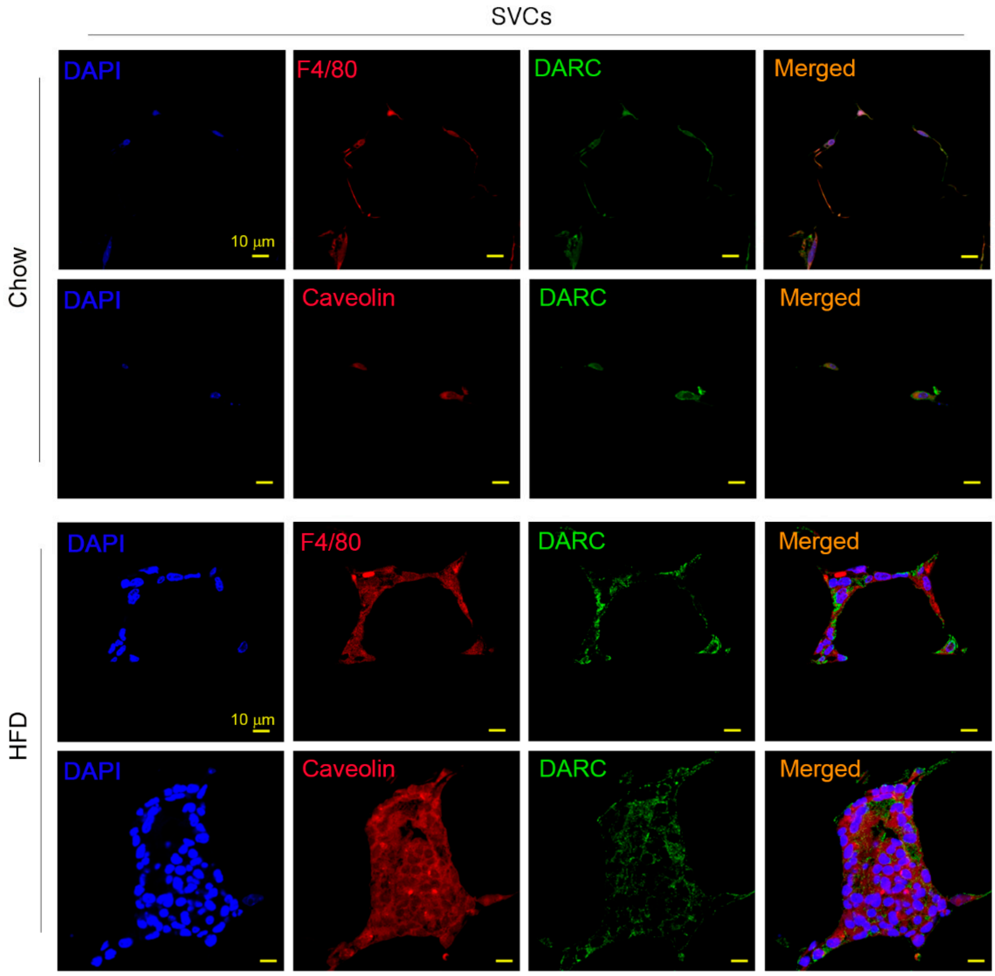

Figure S3

Figure S3. DARC, F4/80, and caveolin expression was determined using immunofluorescent staining. DARC was stained green and monocytes/macrophages (F4/80+) or caveolin red. Nuclei were counterstained with 4',6-diamidine-2'-phenylindole dihydrochloride (DAPI) and the merged image shown indicates the colocalization of the two proteins. Scale bar, 10 μm.

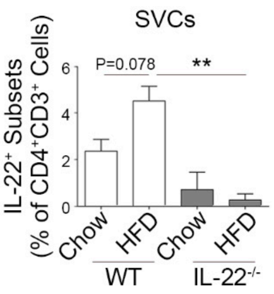

Figure S4

Figure S4. Distribution of the Th22$^+$ subset in eWAT SVCs after HFD consumption. Distribution of the IL-22$^+$ subset was analyzed in the eWAT SVCs of *WT* and *IL-22*$^{-/-}$ mice subjected to a chow diet or HFD. *p < 0.05, **p < 0.001 versus the corresponding control.

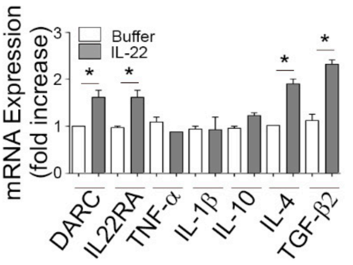

Figure S5

Figure S5. The IL-22 signaling axis enhances M2-like monocyte-derived macrophage potential in the mouse. The effect of IL-22 stimuli on the expression of inflammatory M1- or alternative M2-related genes in BM-derived macrophages was determined by qPCR using the indicated specific primers after treatment with IL-22 for 24 h. *p < 0.05 versus the corresponding control.

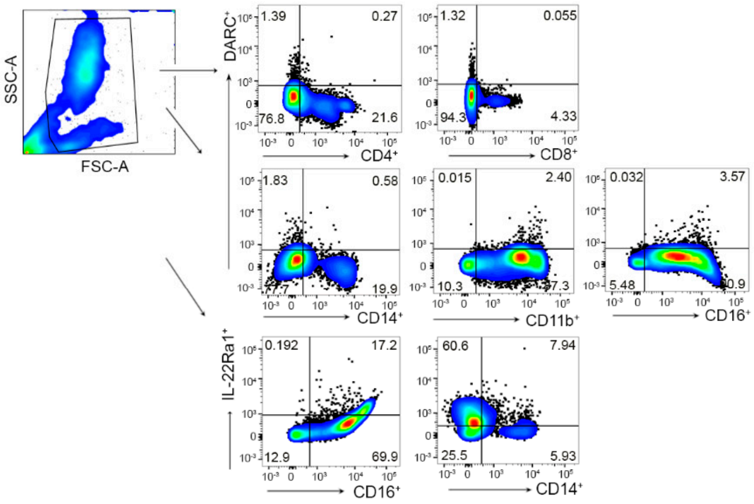

Figure S6

Figure S6. Identification of DARC$^+$ monocytes in human PBMCs. The gating and staining strategy for the quantification of a DARC$^+$ subset was determined by CD4$^+$ or CD8$^+$ (for lymphocytes), and CD14$^+$, CD11b$^+$, or CD16$^+$ (for monocytes/macrophages) cells (upper/middle). The IL22Ra1$^+$ frequency belongs to the CD14$^+$ or CD16$^+$ subpopulation (*lower*).

Supplementary Table I. PCR primers used in this study

Gene name	Primer sequence	
murine GAPDH	Forward	5′-TGGCCTTCCGTGTTCCTAC-3′
	Reverse	5′-GAGTTGCTGTTGAAGTCGCA-3′
murine IL-1β	Forward	5′-AAATACCTGTGGCCTTG-3′
	Reverse	5′-TTAGGAAGACACGGATTC-3′
murine IL-10	Forward	5′-TGGTTTGCCATCGTTTTGCTG-3′
	Reverse	5′-ACAGGTGAGGTTCACTGTTTCT-3′
murine CCL2	Forward	5’-TTAAAAACCTGGATCGGAACCAA-3’
	Reverse	5’-GCATTAGCTTCAGATTTACGGGT-3’
murine CCL5	Forward	5’-GCTGCTTTGCCTACCTCTCC-3’
	Reverse	5’-TCGAGTGACAAACACGACTGC-3’
murine TNF-α	Forward	5'-AGTGACAAGCCTGTAGCC-3'
	Reverse	5'-AGGTTGACTTTCTCCTGG-3'
murine IL-4	Forward	5'-GGTCTCAACCCCCAGCTAGT-3'
	Reverse	5'-GCCGATGATCTCTCTCAAGTGAT-3'
murine IL-12β	Forward	5'-TGGTTTGCCATCGTTTTGCTG-3'
	Reverse	5'-ACAGGTGAGGTTCACTGTTTCT-3'
murine Arg1	Forward	5'-CTCCAAGCCAAAGTCCTTAGAG-3'
	Reverse	5'-AGGAGCTGTCATTAGGGACATC-3'
murine Ym1	Forward	5'-CAGGTCTGGCAATTCTTCTGAA-3'
	Reverse	5'-GTCTTGCTCATGTGTGTAAGTGA-3'
murine iNOS	Forward	5'-GTTCTCAGCCCAACAATACAAGA-3'
	Reverse	5'-GTGGACGGGTCGATGTCAC-3'
murine Fizz1	Forward	5'-CCAATCCAGCTAACTATCCCTCC-3'
	Reverse	5'-ACCCAGTAGCAGTCATCCCA-3'
murine TGFβ2	Forward	5'-AGAATCGTCCGCTTTGATGTC-3'
	Reverse	5'-TCTGGTTTTCACAACCTTGCT-3'
murine DARC	Forward	5'-ATGGGGAACTGTCTGTATCCG-3'
	Reverse	5'-CAGGACACTGGTGAGCATGA-3'
murine IL-22Ra1	Forward	5'-ATGAAGACACTACTGACCATCCT-3'
	Reverse	5'-CAGCCACTTTCTCTCTCCGT-3'
human IL-10	Forward	5'-CCGTGGAGCAGGTGAAGAAT-3'
	Reverse	5'-TATCCCAGAGCCCCAGATCC-3'
human DARC	Forward	5′-CACCCTGATATACAGCACGGA-3′
	Reverse	5′-CCCCATACCCAATGCCTTCT-3′
human IL-22	Forward	5′-GCTTGACAAGTCCAACTTCCA-3′
	Reverse	5′-GCTCACTCATACTGACTCCGT-3′
human IL-22Ra1	Forward	5′-TGTGCCGAGTGAAGACACTG-3′
	Reverse	5′-TGGTGACATATCTGTAGCTCAGG-3′
human IL-1β	Forward	5'-ATGATGGCTTATTACAGTGGCAA-3'

	Reverse	5'-GTCGGAGATTCGTAGCTGGA-3'
human TNF-α	Forward	5'-ATGAGCACTGAAAGCATGATCC-3'
	Reverse	5'-GAGGGCTGATTAGAGAGAGGTC-3'
human TGF-β1	Forward	5'-AAGTGGACATCAACGGGTTC-3'
	Reverse	5'-TGCGGAAGTCAATGTACAGC-3'
human TGF-β2	Forward	5'-CCATCCCGCCCACTTTCTAC-3'
	Reverse	5'-AGCTCAATCCGTTGTTCAGGC-3'
human Arg1	Forward	5'-TGGACAGACTAGGAATTGGCA-3'
	Reverse	5'-CCAGTCCGTCAACATCAAAACT-3'
human CD86	Forward	5'-CTGCTCATCTATACACGGTTACC-3'
	Reverse	5'-GGAAACGTCGTACAGTTCTGTG-3'
human CD206	Forward	5'-TCCGGGTGCTGTTCTCCT-3'
	Reverse	5'-CCAGTCTGTTTTTGATGGCACT-3'
human CD163	Forward	5'-GACGCATTTGGATGGATCATGT-3'
	Reverse	5'-CCCACCGTCCTTGGAATTTGA-3'
human GAPDH	Forward	5′TGTTGCCATCAATGACCCCTT-3′
	Reverse	5´-CTCCACGACGTACTCAGCG-3´
